# Microstructures and mechanical properties of *in situ* TiC–β–Ti–Nb composites with ultrafine grains fabricated by high-pressure sintering

**DOI:** 10.1038/s41598-018-27535-6

**Published:** 2018-06-22

**Authors:** Z. Liu, D. C. Zhang, L. J. Gong, J. G. Lin, Cuie Wen

**Affiliations:** 1 School of Materials Science and Engineering, Xiangtan University, Xiangtan, 411105 Hunan China; 2 Key Laboratory of Materials Design and Preparation Technology of Hunan Province, Xiangtan University, Xiangtan, 411105 Hunan China; 3 School of Engineering, RMIT University, Victoria, 3083 Australia

## Abstract

In this study, an *in situ* β–Ti–Nb composites reinforced with TiC particles with an ultrafine grain size were fabricated using a powder metallurgical (PM) method. The microstructures and mechanical properties of the composites were characterized using X-ray diffraction (XRD) analysis, scanning electron microscopy (SEM), transmission electron microscopy (TEM) and compression tests. TiC particles were formed in the ball-milled powders after annealing at 600 °C due to a chemical reaction between stearic acid and titanium. Using high-pressure sintering (HPS) on an apparatus with six tungsten carbide anvils, a fully dense β–Ti–Nb composite reinforced with fine *in situ* TiC particles was obtained. The TiC particles exhibit particle sizes of ~500 nm, uniformly distributed in the composite matrix, which had grain sizes of ~600 nm. Thus, the TiC–β–Ti–Nb composite show very high compression yield strength and relatively high plasticity contributed by grain refinement and TiC particles strengthening. The composite with 45 vol.% TiC exhibited excellent mechanical properties, with a yield compressive strength of 1990 MPa and plastic strain of 9.12%. More over, a modified rule-of-mixture (ROM) was presented to describe the combined strengthening effect of grain refinement and TiC particles.

## Introduction

Titanium (Ti) and its alloys are widely used in aerospace, automotive, medical device, and other industries due to their low density, high specific strength, high temperature properties, and excellent biocompatibility^1,2^. To improve the mechanical properties of Ti alloys, a series of Ti alloy matrix composites (TMCs) have been developed which exhibit higher specific strength, specific stiffness, wear resistance, thermal stability, and high-temperature durability than conventional Ti alloys^3^. Normally, TMCs are produced by *ex situ* techniques in which the reinforcements are prepared separately prior to the composite fabrication^4,5^, while *in situ* techniques synthesize the reinforcements during the fabrication of the composites^6–11^. Compared to *ex situ* TMCs, *in situ* TMCs exhibit finer grain sizes, more homogeneous distribution of reinforcements, and cleaner interfaces, leading to enhanced mechanical properties. It has been reported that the powders prepared by high-energy ball milling, mixing Ti powder with process control agents such as toluene^12^, n*-*heptane^13^, and stearic acid (SA)^14^, can *in situ* synthesize non-stoichiometric TiC_x_ and TiH_x_ in the subsequent annealing or sintering, and the TiH_x_ will desorb hydrogen at further elevated temperatures. This approach offers the potential for preparing *in situ* TiC-reinforced TMCs. This approach, rather than choosing graphite^15^ or B_4_C^16^ as the carbon (C) source, can effectively reduce agglomeration and decrease the size of particles, to offer the potential for preparing *in situ* TiC-reinforced TMCs.

It has been well documented that powder metallurgy products with fine grain size and low porosity possess good mechanical properties^17,18^. However, consolidation of powders into a fully dense solid with nano-scale grain size is a major challenge for industrial applications. Conventional sintering production has some drawbacks, such as long sintering time, low density, and coarse grain, leading to poor mechanical properties. It was reported that sintering ultrafine powders under high pressure may achieve almost fully dense materials with ultrafine grain size, because the applied high pressure could provide extra driving force for densification, promote nucleation, and reduce the overall growth rate of grains^19^. Using this method, many materials with ultrafine grains have been developed. For example, Liao *et al*.^19^ successfully prepared nanocrystalline TiO_2_ materials with almost full density and a grain size less than 75 nm. He *et al*.^20^ prepared almost fully dense Fe_3_Al and Ni_3_Al with a grain size below about 20 nm via hot pressing/forging methods. Moreover, some composites have also been prepared using hot pressing under high pressures, such as aluminum (Al) matrix composites reinforced by Al_60_Cu_20_Ti_15_Zr_5_ glassy particles^21^ and TMCs reinforced by *in situ* TiC particles with nano-scale sizes^22^.

In this study, we used elemental titanium (Ti) and niobium (Nb) powders as raw materials. The powders, mixed with stearic acid (SA) as process control agent and a carbon (C) source, were high-energy ball-milled and then annealed at 600 °C. Finally, the powders were sintered under high pressure using an apparatus with six tungsten carbide anvils. The microstructures and mechanical properties of the composites were evaluated, aiming to develop a TiC–β–TiNb composite with enhanced mechanical properties for industrial applications.

## Results

### Microstructures

**Figure 1:**
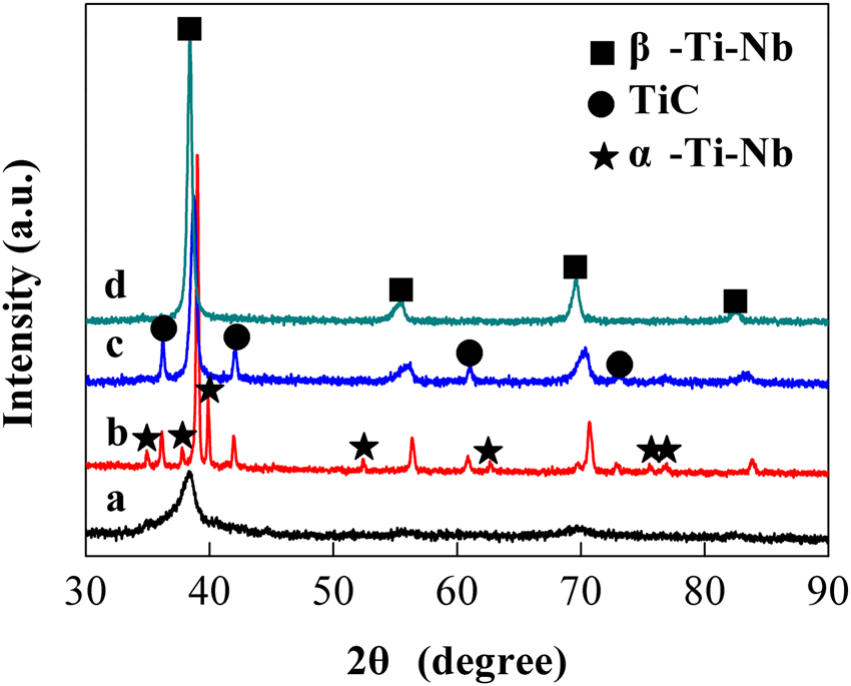
XRD patterns of (**a**) ball-milled Ti–32 Nb powders with 2.4 wt.% SA addition; (**b**) annealed Ti–32 Nb powders with 2.4 wt.% SA addition; (**c**) as-sintered sample consolidating the annealed Ti–32 Nb powders with 2.4 wt.% SA addition; and (**d**) as-sintered Ti–32 Nb sample.

Figure 1 shows the XRD patterns of the Ti–32 Nb powders with 2.4 wt.% SA addition ball-milled for 10 h, the powders annealed at 600 °C for 2 h, and the as-sintered sample consolidating the annealed powders at 1200 °C for 15 min under 3 GPa. For comparison, the XRD pattern of the as-sintered Ti–32 Nb alloy prepared under the same conditions is also illustrated in Fig. 1. From the figure, it can be seen that the XRD pattern of the ball-milled powders with 2.4 wt.% SA addition only consists of the diffraction peaks from the β–Ti–Nb phase, implying that complete mechanical alloying of Ti–Nb occurred after ball milling for 10 h, and the average grain size of the ball-milled powders was estimated to be 19.7 nm according to the Scherrer formula^23^. After annealing at 600 °C for 2 h, the diffraction peaks from TiC appeared on the XRD pattern of the Ti–32 Nb powders with 2.4 wt.% SA addition. This indicates the formation of TiC during the powder annealing at 600 °C. The annealed Ti–32 Nb powders with 2.4 wt.% SA addition were consolidated at 1200 °C under a high pressure of 3 GPa. The phase constitutions of the as-sintered samples were determined by the XRD method and the result is illustrated in Fig. 1c. It can be seen that the as-sintered sample consists of two phases, β–Ti–Nb and TiC. The results indicate that SA can be used as a C source, which can react with Ti during the ball-milled powder annealing at 600 °C to produce TiC *in situ* in the alloy powders. To further confirm this, a Ti–32 Nb alloy without SA addition was prepared under the same conditions as the alloy with SA addition. The XRD analysis result shows that the as-sintered Ti–32 Nb alloy only consists of the β–Ti–Nb phase (see Fig. 1d).

**Figure 2:**
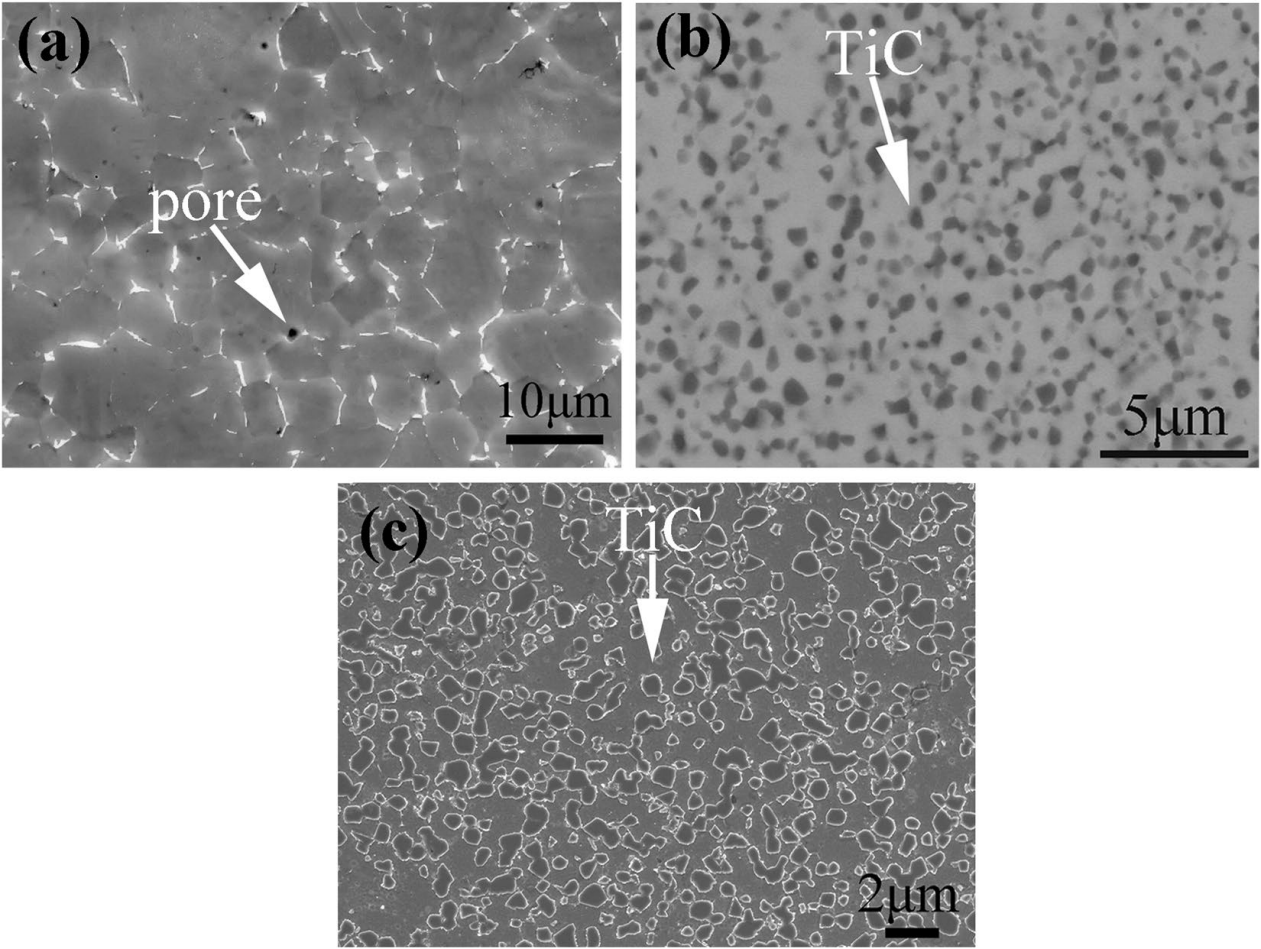
(**a**) SEM secondary electron image of as-sintered Ti–32 Nb sample; (**b**) SEM backscattered electron image of as-sintered 35 vol.% TiC–β–Ti–Nb composite; and (**c**) SEM secondary electron image of as-sintered 45 vol.% TiC–β–Ti–Nb composite.

The microstructures of the as-sintered TiC–β–Ti–Nb composites were characterized using SEM in comparison with the Ti–32 Nb alloy. Figure 2 shows SEM images of the microstructures of the Ti–32 Nb, Ti–32 Nb with 1.8 wt.% SA addition, and Ti–32 Nb with 2.4 wt.% SA addition samples, respectively. From the secondary SEM image of the as-sintered Ti–32 Nb alloy (Fig. 2a), one can see that it contains β phases with a grain size of about 6 μm, and some impurity phases in bright contrast and small pores existing at the grain boundaries. The impurity phase contains Ti, Nb and Fe, in which Fe element comes from steel ball grinding tube during milling process. By the Archimedes method, the density of the alloy is about 96%, indicating that full densification has not been achieved in the alloy. By contrast, the microstructures of the as-sintered samples of Ti–32 Nb with 1.8 wt.% SA addition and Ti–32 Nb with 2.4 wt.% SA addition exhibit a large number of ultrafine TiC particles with sizes ranging from 200 nm to 800 nm, homogeneously dispersed in the matrix, and the volume fractions of the TiC particles for Ti–32 Nb with 1.8 wt.% SA addition and Ti–32 Nb with 2.4 wt.% SA addition are 35 vol.% and 45 vol.%, respectively. No pores can be observed in these two alloys and their density was measured to be about 99%, implying that full densification has almost been achieved in the two alloys.

**Figure 3:**
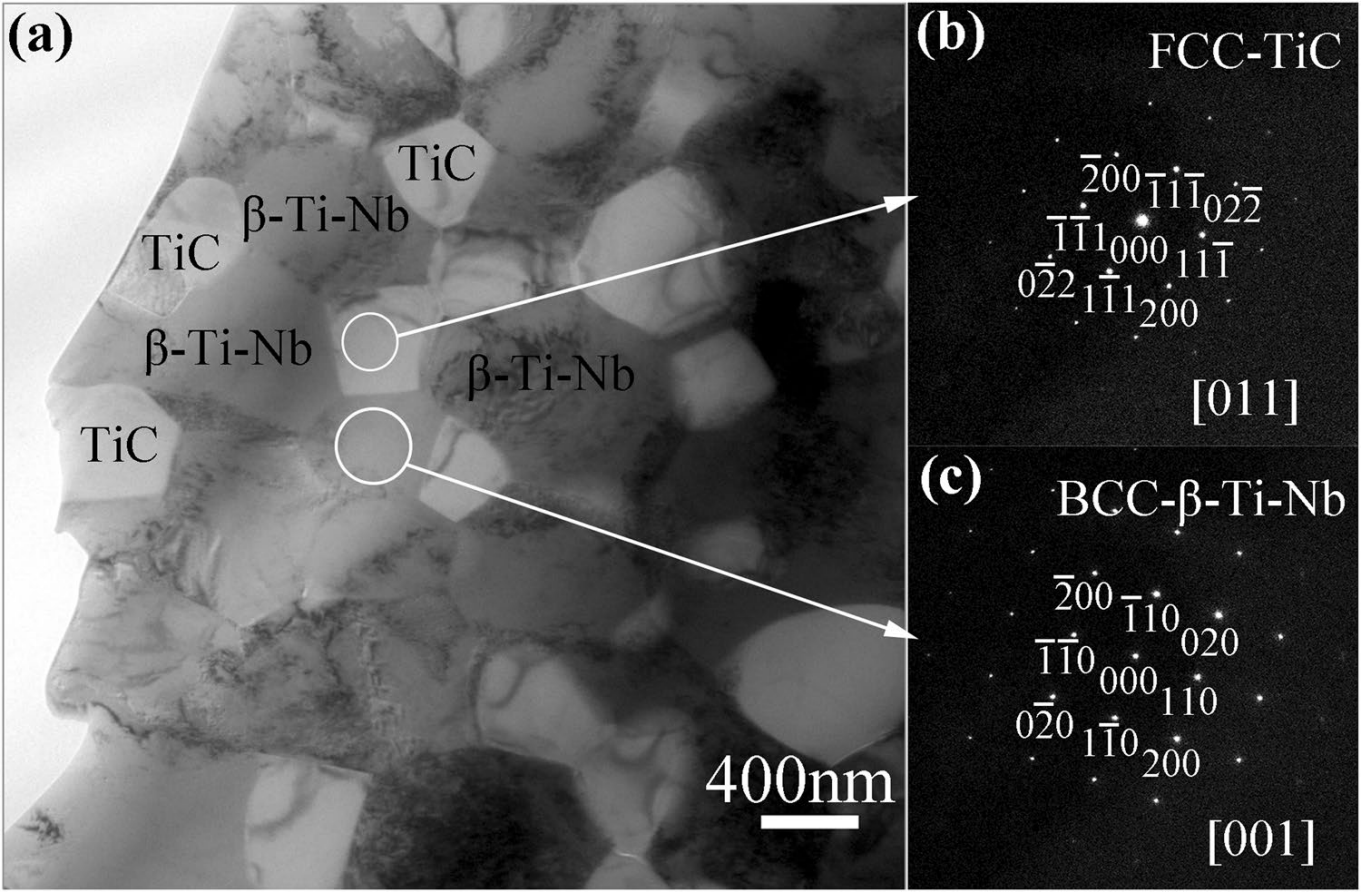
(**a**) Bright-field TEM image of as-sintered 45 vol.% TiC–β–Ti–Nb composite; (**b**) SAED pattern taken along [011] zone axis of one of the second phase particles, corroborating that the second phase is FCC–TiC; and (**c**) SAED pattern taken along [001] zone axis of the matrix, corroborating that the matrix is BCC–β–Ti–Nb.

Furthermore, close observations were conducted of the 45 vol.% TiC–β–Ti–Nb composite on a TEM. Figure 3 shows a bright-field TEM image of the as-sintered 45 vol.% TiC–β–Ti–Nb composite and the corresponding SAED patterns. It can be seen that the as-sintered TiC–β–Ti–Nb composite contains two phases (see Fig. 3a). The bright phases are determined to be TiC with an FCC structure, while the dark phases are β–Ti–Nb phases with a BCC structure, according to the corresponding SAED patterns (see Fig. 3b and c). The TiC particles have a grain size of ~500 nm and the matrix has a grain size of ~600 nm, which illustrates that the HPS sample with SA addition is a TiC–β–Ti–Nb ultrafine grain composite. These results are in good agreement with those of the XRD analysis and SEM observations.

### Mechanical properties

**Figure 4:**
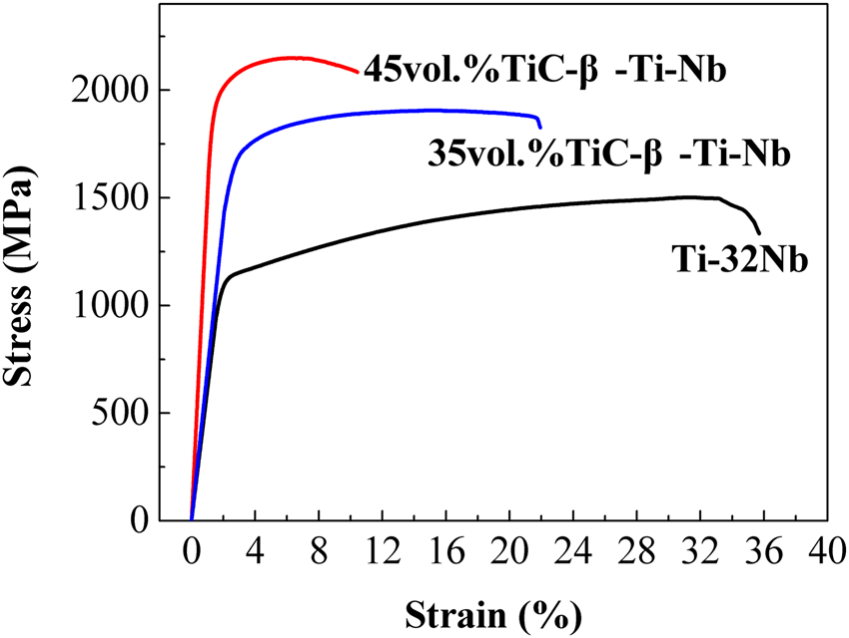
Room-temperature compressive stress–strain curves of as-sintered samples.

The mechanical compression properties of the 35 vol.% TiC–β–Ti–Nb and 45 vol.% TiC–β–Ti–Nb composites were evaluated in comparison with that of the Ti–Nb alloy prepared under the same conditions. Figure 4 shows the room-temperature compressive stress–strain curves of all the samples after consolidation. It can be seen that the as-sintered β–Ti–32 Nb alloy exhibits a high yield strength of 1100 MPa with a large plastic strain of 32.55%, while for the two composites of 35 vol.% TiC–β–Ti–Nb and 45 vol.% TiC–β–Ti–Nb, a distinct increase in yield strength has been achieved due to the presence of *in situ* TiC reinforcements, and their yield strengths are 1710 MPa and 1990 MPa, respectively. Moreover, the two composites also exhibit a certain plastic strain, which reaches 17.42% and 9.12%, respectively.

**Figure 5:**
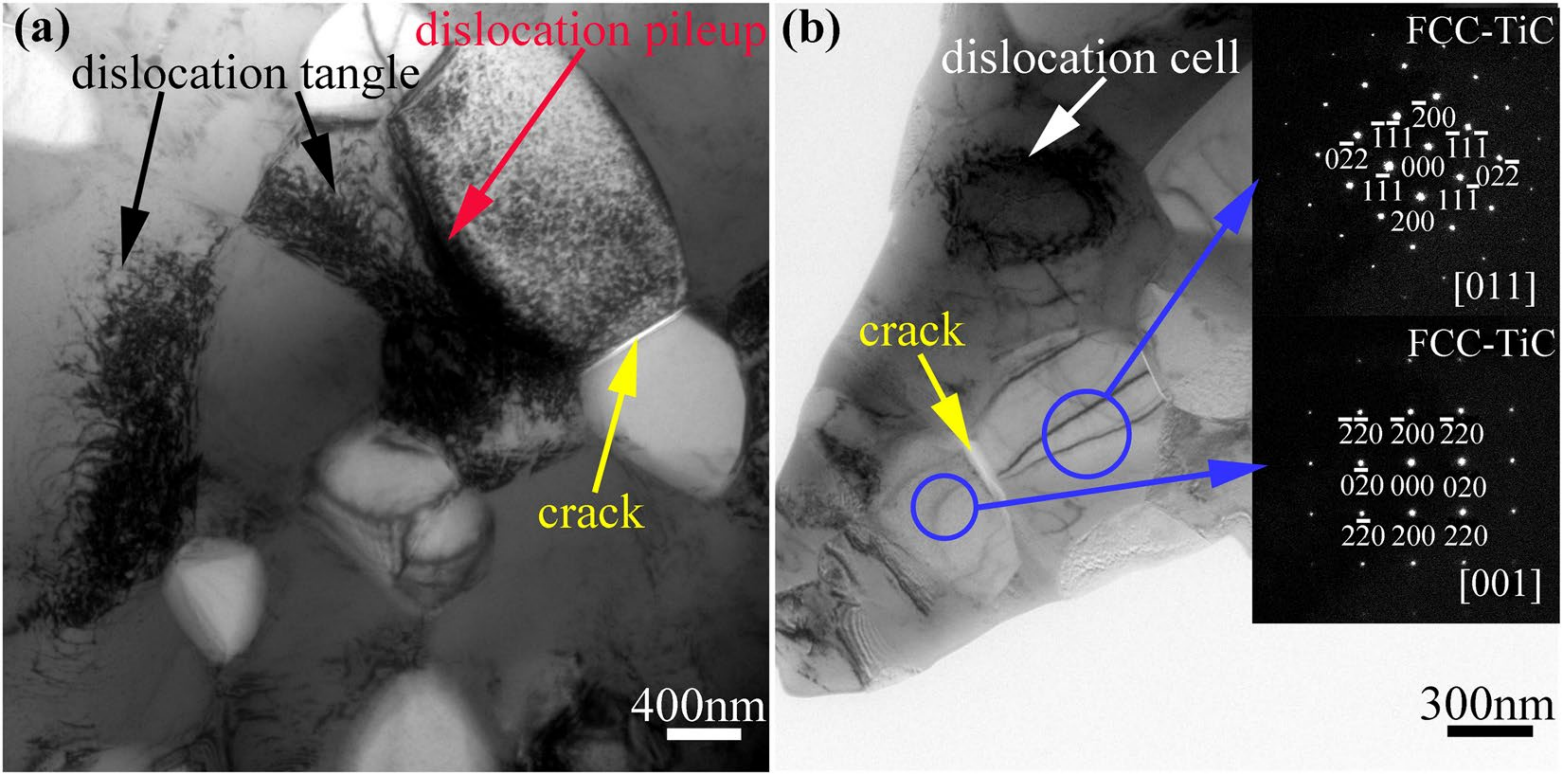
TEM micrographs of partially deformed (strain = 8%) 45 vol.% TiC–β–Ti–Nb ultrafine grain composite, presenting dislocation tangle (marked by black arrows in image (**a**)), dislocation pileup (marked by red arrow in image (**a**)), dislocation cell (marked by white arrow in image (**b**)) in β–Ti–Nb matrix, and cracks (marked by yellow arrows in both image (**a**) and (**b**)) between TiC particles, confirmed by SAED patterns.

To illustrate the plastic deformation mechanisms of the TiC-particle-reinforced β–Ti–Nb composites prepared in the present work, the deformation microstructure of the 45 vol.% TiC–β–Ti–Nb composite was observed. Figure 5 shows TEM images of the 45 vol.% TiC–β–Ti–Nb composite after a deformation of 8%. It is clear that the high density of dislocations in the β–Ti–Nb grains hindered the interfaces between the TiC particles and the matrix, which interacted to form the dislocation tangle, the dislocation wall, and the dislocation cells, implying that severe deformation occurred in the β–Ti–Nb grains. In contrast, few dislocations can be seen in the TiC grains. However, some cracks can be observed at the grain boundaries of TiC particles. So, during the compressive deformation of the composite, the plastic deformation first occurred in the matrix as the external load exceeded the yield strength of β–Ti–Nb. With the external load further increasing, the matrix hardened and the TiC particles were the load-bearing elements, leading to the increase in the yield strength of the composite. When the stress concentration in the particles due to dislocation pill-up exceeded the strength of the TiC, cracks emerged at the grain boundaries of the TiC grains, rather than at the interfaces between TiC and β–Ti–Nb, which implies that the composites created strong interfaces between TiC and β–Ti–Nb, and the failure occurred at the grain boundaries of the TiC particles.

**Figure 6:**
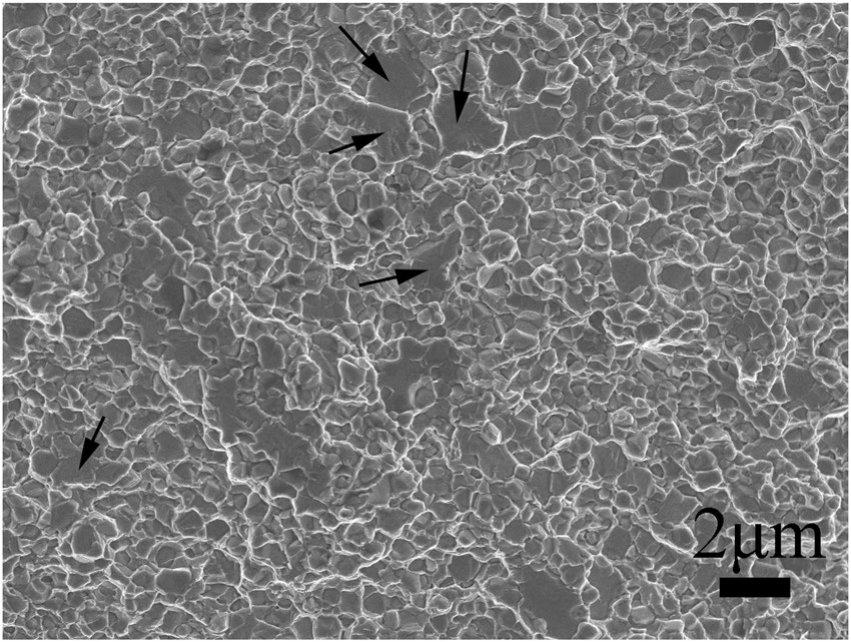
SEM image of fracture surface of as-sintered 45 vol.% TiC–β–Ti–Nb ultrafine grain composite. Black arrows show TiC particles plain fractures surrounded by β–Ti–Nb matrix dimpled fractures.

Figure 6 shows a SEM micrograph of the fracture surface of the 45 vol.% TiC–β–Ti–Nb composite. It is clear that the fracture surface exhibits a rather rough characteristic, indicating a complex crack propagation, while the TiC reinforcements show plain fractures surrounded by matrix dimples. This means the fractures of this composite have mixed fracture characteristics of dimples and intergranular cracking.

## Discussion

It has been well documented that solid state reactions via mechanical alloying (MA) can take place due to the negative heat of mixing and these reactions proceed via the interdiffusion of the components into thin multilayers, and a large amount of lattice defects and interfaces introduced during milling may be conducive to the solid state reactions [12]. So it has been reported that, by MA methods, metastable Ni_3_C can be obtained in Ni–graphite powder mixtures^24^, NbC in Cu-Nb-graphite mixtures^25^, and TiC in Ti–n-heptane mixtures^12^. Thus, the process of formation of TiC in the Ti–Nb alloy powders during ball milling and subsequent annealing in the present work can be described as follows: In the initial period of mechanical alloying, SA melts and evenly adsorbs at the surfaces of the Ti and Nb powders, with many defects introduced by ball milling. With ball-milling time increasing, the size of the powders decreases and Nb gradually dissolves into Ti to form a β–Ti–Nb solid solution. Most of the SA molecules enrich the grain boundary. In the subsequent annealing process, the Ti–Nb alloy powders with nano-size particles and many defects easily react with the SA molecules, and the possible reactions are as follows:

1 $${\rm{TiC}}+{\rm{C}}={\rm{TiC}}$$

2 $${\rm{Nb}}+{\rm{C}}={\rm{NbC}}$$

3 $${\rm{Ti}}+{\rm{2H}}={{\rm{TiH}}}_{2}$$

The corresponding Gibbs energy for the three possible reactions at different temperatures can be calculated according to

4 $${\rm{\Delta }}{G}_{f,B}(T)={G}_{B}(T)-|\sum {v}_{{\varepsilon }_{i}}|{G}_{{\varepsilon }_{i}}({T})\,({\rm{KJ}}/{\rm{mol}}),$$

where ΔG_*f,B*_(*T*) is Gibbs energy of reaction for the formation of *B* from the elements *ε*_*i*_, G_*B*_(*T*) is Gibbs energy of *B*, ${G}_{{\varepsilon }_{i}}(T)$ is Gibbs energy of the element *ε*_*i*_, which are listed in Table 1^26^. It is clear that the reaction (1) yield more negative Gibbs free energy change, which implies it is easier to occur and the reaction products of TiC are more stable than NbC. So, we can only observe the presence of the TiC particles in the annealed and as-sintered samples. As for the TiH_x_ generated during low-temperature annealing, it desorbs hydrogen at further elevated temperatures under vacuum^13^.

**Table 1:** Gbbis energy (KJ/mol) of different reactions at different temperatures (K).

	ΔG_*f*_ (600)	ΔG_*f*_ (900)	ΔG_*f*_ (1200)	ΔG_*f*_ (1500)
TiC	−177.336	−174.179	−170.773	−166.292
NbC	−135.371	−134.440	−133.717	−133.103
TiH_2_	−63.188	−20.651	21.585	64.148

The TiC–β–Ti–Nb composites prepared in the present work exhibits the full dense and the ultrafine microstructures. It may be attributed to the high sintering pressure and the presence of the nano-scale TiC particles *in-situ* produced in the annealing and sintering process. It is well documented the applied high pressure during sintering could provide extra driving force for densification, promote nucleation, and reduce the overall growth rate of grains^19^. Moreover, the fine TiC particles replace part of the grain boundaries, which may act as the barriers of the grain growth^27^. According to the Eq. 5^28^,

5 $${\lambda }_{m}=4(1-f\,){r}_{p}/{\rm{3}}{f}$$

where λ_*m*_, *r*_*p*_ and *f* correspond to the distance apart from the reinforcements, the radius of the particles and the fractional volume of reinforcements, respectively. It can be deduced that the grain size of matrix decreases with the increase of the fractional volume of reinforcements, which is evidenced by the grain sizes of the samples with 35, and 45% TiC particles.

The TiC–β–Ti–Nb composites prepared in the present work exhibit the ultrahigh strength, which reaches 1990 MPa for the sample with 45 vol.% TiC particles, and it arises from the grain refinement and the reinforcement of the TiC particles. So, a modified rule-of-mixture (ROM)^29^ can be described as

6 $${\sigma }_{c}=({\sigma }_{m}+{\rm{\Delta }}{\sigma }_{{gr}}{)V}_{m}+{\sigma }_{{TiC}}{V}_{{TiC}}$$

where *σ*_*c*_ is the strength of the TiC–β–Ti–Nb composites, *σ*_*m*_ is the strength of β–Ti–Nb matrix, Δ*σ*_*gr*_ is the strength increment arising from the grain refinement, *σ*_*TiC*_ is the strength of TiC particles, *V*_*m*_ is the volume fraction of β–Ti–Nb matrix, and *V*_*TiC*_ is the volume fraction of TiC particles. The strength contributed by the grain refinement can be estimated by Hall-Patch relationship^30^,

7 $$\tau ={\tau }_{0}+k{d}^{-1/2}$$

where *τ* is the yield stress, *τ*_0_ is the friction stress needed to move individual dislocations, *k* is a constant, and *d* is the average grain size.

Assuming *d*_*o*_ is the average grain size of β–Ti–Nb matrix without TiC particles and *d*_*gr*_ is average grain size of β–Ti–Nb matrix refined by TiC particles. The yield stress of the β–Ti–Nb matrix without TiC particles, *τ*_*o*_, can be described as

8 $${\tau }_{o}={\tau }_{0}+{k}{d}_{o}^{-1/2}$$

and the yield stress of β–Ti–Nb matrix refined by TiC particles

9 $${\tau }_{{gr}}={\tau }_{0}+{k}{{d}}_{{gr}}^{-1/2}$$

Therefore, the β–Ti–Nb matrix strength increment contributed by the grain refinement can be estimated by

10 $${\rm{\Delta }}{\sigma }_{{gr}}={\tau }_{{gr}}-{\tau }_{o}={k}({d}_{{gr}}^{-1/2}-{d}_{o}^{-1/2})$$

where *k = *0.4 MN/m^3/2 ^^31^. Thus, as the grain size of the matrix decrease from 6 μm to 1.2 μm and 0.6 μm, the increments of the strength are estimated to be about 202 MPa and 353 MPa, according to Eq. 10.

Assuming *σ*_*TiC*_ = 2600 MPa for C/Ti = 0.6~0.8^32^ (averaging the strength of TiC), we can estimated the strength for the composites with 35 vol.% and 45 vol.% TiC particles to be about 1756 MPa and 1969 MPa, according to Eqs 6 and 10, respectively. As the result, the theoretically predicted yield strength of the as-sintered samples considering the grain refinement and the second phase reinforcement can be obtained, which are listed in Table 2, in comparison with the experimental results. It is clear that the theoretical results are in a good agreement with the experimental ones.

**Table 2:** The predicted and experimental strength for as-sintered samples.

As-sintered samples	Strength of the β–Ti–Nb matrix, *σ*_*m*_ (MPa)	Grain size of β–Ti–Nb matrix, *d*_*gr*_ (μm)	Strength increment contributed by grain refinement, Δ*σ*_*gr*_ (MPa)	Theoretical strength, *σ*_*c*_ (MPa)	Yield strength (MPa)
β–Ti–Nb	1100	6	—	—	1100
35 vol.% TiC–β–Ti–Nb	1100	1.2	202	1756	1710
45 vol.% TiC–β–Ti–Nb	1100	0.6	353	1969	1990

## Conclusions

TiC-β-Ti-Nb composites with fully dense and ultrafine grain were successfully prepared by HPS. The microstructure and mechanical properties of the as-sintered samples were studied, and the following conclusions can be drawn:

1. TiC particles on nano-scales were synthesized *in situ* in the powders due to reactions between Ti and C. The elemental powders of Ti and Nb mixed with stearic acid were ball milled on a high-energy planetary miller. Complete mechanical alloying of the Ti–Nb powder mixtures was achieved after ball milling for 10 h, and β–Ti–Nb alloy powders were obtained. After the ball-milled powders were annealed at 600 °C for 2 h.
2. The composites were almost fully dense, with a density of 99%, and retained ultrafine grains, with grain sizes of ~500 nm for the reinforcements (TiC) and ~600 nm for the matrix. By sintering of the annealed alloy powders at 1200 °C under a high pressure of 3 GPa for 15 min, a β–Ti–Nb alloy matrix with ultrafine grains reinforced by *in situ* TiC particles was successfully fabricated.
3. The composites exhibited high compressive yield strength and relatively high plasticity, and the ultrahigh strength of the composite arise from the grain refinement and the TiC particle reinforcement. For the 35 vol.% TiC–β–Ti–Nb and 45 vol.% TiC–β–Ti–Nb composites, the yield strengths were 1710 MPa and 1990 MPa, and the plastic strain were 17.42% and 9.12%, respectively.
4. A new rule-of-mixture (ROM) was presented to describe the combined strengthening effect of grain refinement and TiC particles. The modified ROM can be described as ${\sigma }_{c}=({\sigma }_{m}+{\rm{\Delta }}{\sigma }_{{gr}}){V}_{m}+{\sigma }_{{TiC}}{V}_{{TiC}}$.

## Methods

**Figure 7:**
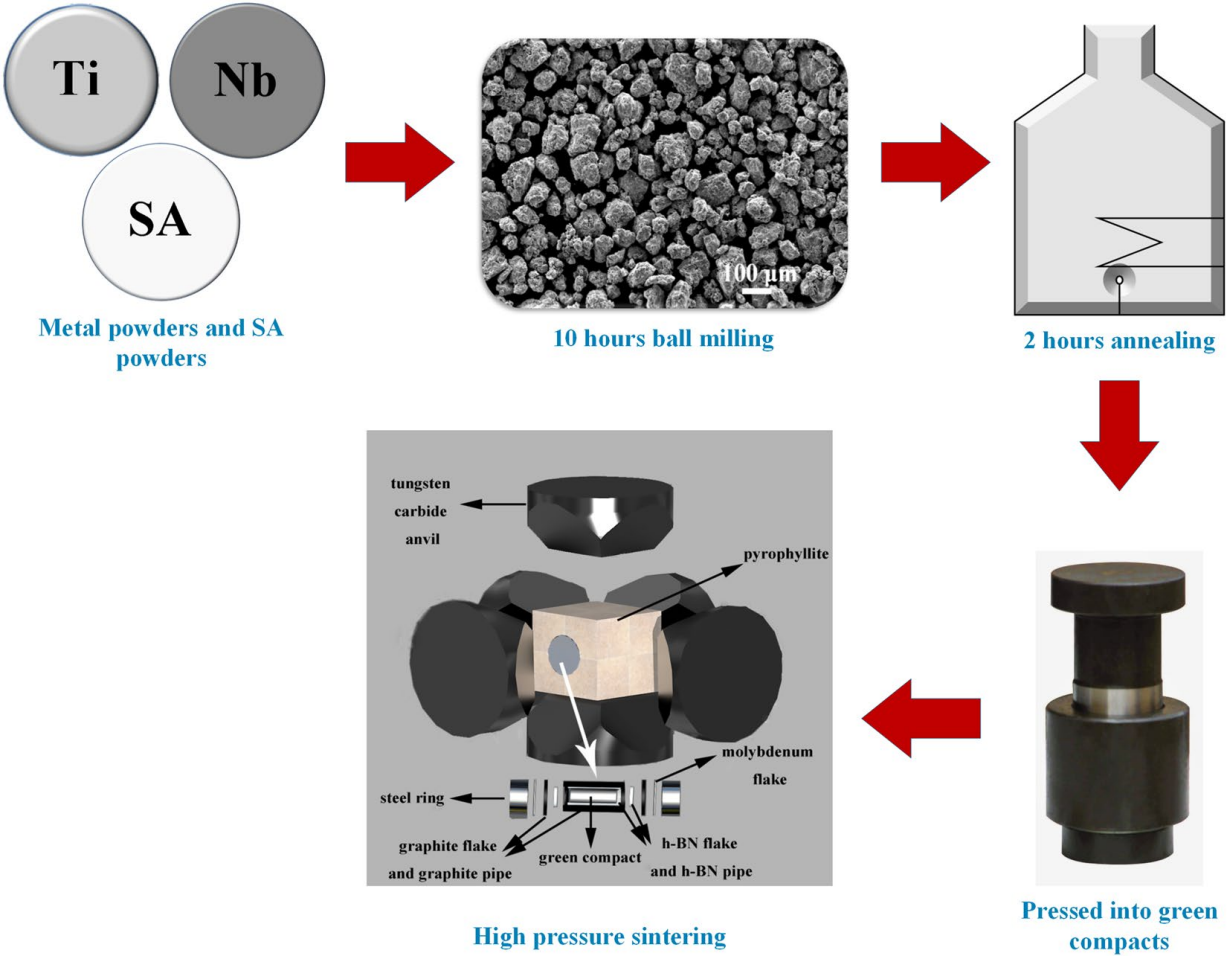
Preparation flow chart for TiC-β-Ti-Nb composites.

TiC-β-Ti-Nb composites preparation flow chart is presented in Fig. 7. Elemental powders of Ti and Nb with a purity of 99.9% and average particle size smaller than 50 μm were mixed in the desired composition of Ti_68_Nb_32_ (in weight percent, wt.%). To decrease the agglomeration of the powders during ball milling, SA powders were added into the mixed powders. The mixtures were placed into a 250 ml stainless steel vial together with stainless steel balls with a diameter of 10 mm under vacuum. The ball-to-powder weight ratio was selected as 15:1. Mechanical alloying was performed at a speed of 200 rpm on a planetary ball mill (QM-2SP2; apparatus factory of Nanjing University, PR China). After ball milling for 10 h, the Ti–Nb powders with SA addition were annealed at 600 °C for 2 h under vacuum of 6.67 × 10^−3^ Pa.

Subsequently, the alloy powders were pressed into green compacts with a cylindrical shape of 10 mm in diameter and 8 mm in height using a tablet press for high-pressure sintering (HPS). The HPS process was performed on an apparatus with six tungsten carbide anvils. A schematic sample assembly for the HPS experiment is shown in Fig. 7. Pyrophyllite was chosen as the pressure medium. The heating was performed by passing electric current through the graphite crucible containing the insulating layer and the sample. The samples were first pressed at 3 GPa, and then heated to 1200 °C at a rate of approximately 300 K/s and held for 15 min. After that, the samples were cooled down to room temperature by switching off the heater under the pressure. Finally, the samples were taken out for testing after pressure relief.

The densities of the as-sintered samples were measured by the Archimedes method. X-ray diffraction (XRD) patterns were taken with an Ultima IV X-ray diffractometer with Cu Ka radiation for the powders after milling and annealing, and after consolidation. A JEOL-JSM6700F scanning electron microscope (SEM) with energy dispersive spectroscopy (EDS) and a JEOL-JEM2100 transmission electron microscope (TEM) with EDS (both JEOL, Tokyo, Japan) operated at 200 kV accelerating voltage were used for characterization of the microstructures of the as-sintered samples. Compression properties were tested by using an Instron 5569 (Instron, Massachusetts, USA) testing machine at a strain rate of 1 × 10^-4^ s^-1^ at room temperature. The compression samples were cut into a cylindrical shape with a dimension of 3 mm in diameter and 6 mm in height according to ASTM standards.
